# Disseminated endometrial tuberculosis simulating endometrial cancer in an immunocompetent patient: a case report

**DOI:** 10.17843/rpmesp.2026.432.15677

**Published:** 2026-06-24

**Authors:** Juana V. Vera-Vera, Cristhian K. Landa-Huaman, Melissa A. Romero-Aliaga, Fernando R. Garayar-Burneo

**Affiliations:** 1 Hospital Nacional Edgardo Rebagliati Martins, Lima, Peru.

**Keywords:** Female Genital Tuberculosis, Uterine Hemorrhage, Postmenopause, Endometrial Neoplasms, Differential Diagnosis, Pulmonary Tuberculosis

## Abstract

The case of a 58-year-old immunocompetent woman with no relevant medical history, presenting with pelvic pain and postmenopausal bleeding, is reported. Imaging studies revealed findings compatible with disseminated endometrial cancer, including pulmonary lesions suspicious for metastasis, for which advanced gynecological cancer was initially presumed; however, endometrial biopsy showed necrotizing granulomas with acid-fast bacilli, confirming endometrial tuberculosis (TB). Likewise, pulmonary TB involvement was evidenced (positive sputum smear), establishing the final diagnosis of disseminated TBsimulating a gynecological cancer. The patient received standard anti-TB treatment for six months, with complete remission of symptoms. A post-treatment follow-up biopsy was negative for TB. This case emphasizes the importance of considering genital TB in the differential diagnosis of gynecological neoplasms, even in immunocompetent postmenopausal patients, especially in TB-endemic regions, to ensure timely diagnosis and management.

## INTRODUCTION

Extrapulmonary tuberculosis (TB) constitutes approximately 10 to 20% of TB cases in immunocompetent patients. This proportion significantly increases in the presence of immunosuppression, reaching over 60% in cases of HIV coinfection. Among extrapulmonary forms, lymph node involvement is the most common, followed by genitourinary TB ^1^^-^^3^.

Female genital tuberculosis (FGT), although uncommon in developed countries, continues to be a significant cause of gynecological morbidity in developing countries, and is a relevant diagnosis due to its clinical implications, including secondary infertility, chronic pelvic pain, menstrual disorders, and nonspecific symptoms such as postmenopausal bleeding ^(^^4^^,^^5^.

FGT generally originates from the hematogenous dissemination of *Mycobacterium tuberculosis* from a primary pulmonary focus to the female reproductive organs. The most affected sites are the fallopian tubes in almost all cases, 90 to 100%, and the endometrium in 50 to 80% of patients. It is less common for the infection to affect the ovaries, cervix, or vagina ^4^^,^^6^. Although it is more common in young women of reproductive age, the disease can manifest at any age, even in postmenopausal women previously exposed to primary tuberculous infection in early stages of their lives. In older immunocompetent patients, FGT is usually related to the endogenous reactivation of a latent infection acquired during youth ^(^^7^.

Clinical presentation is often nonspecific and insidious, significantly hindering timely diagnosis. For this reason, diagnosis is often established incidentally during infertility evaluations, or during diagnostic investigation of clinical conditions that simulate gynecological malignant processes ^4^. In fact, a relevant clinical feature is the ability of FGT to simulate gynecological neoplasms, especially endometrial or ovarian cancer, given its clinical and imaging presentation with adnexal masses, endometrial thickening, peritoneal carcinomatosis, and ascites ^4^^,^^6^^,^^8^. This diagnostic confusion can lead to a significant delay in appropriate management, increasing the morbidity associated with this entity.

Another important aspect to consider is the occurrence of FGT in immunocompetent patients. Although disseminated or extrapulmonary TB is classically associated with marked immunodeficiency states (HIV, prolonged immunosuppressive treatment, among others), approximately 10 to 20% of extrapulmonary cases occur in individuals without evident immunosuppression factors ^3^^,^^9^. Therefore, the absence of immunosuppression should not exclude the diagnostic suspicion of genital TB, particularly in endemic areas.

A clinical case of disseminated endometrial TB in an immunocompetent patient, with an initial diagnosis of advanced endometrial cancer, is described. The diagnostic and therapeutic challenges of this rare clinical entity are discussed, highlighting the importance of early histological diagnosis and the clinical relevance of considering this disease within the differential diagnosis in patients with an initial suspicion of malignant gynecological neoplasm.

## CASE REPORT

### Patient Information

**Figure 1:**
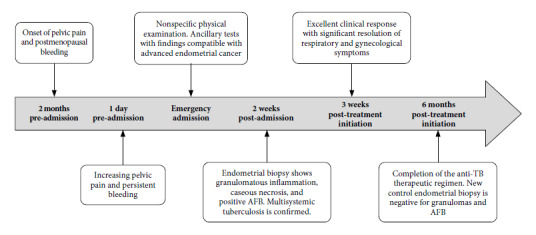
Timeline of the chronological narrative.

A 58-year-old female patient, native and resident of the district of Lince in Lima, with a gynecological-obstetric history of two full-term deliveries (G2P2002), menopause established eight years ago with no prior history of menstrual alterations, and no relevant pathological history, who attended the consultation for persistent pelvic pain and postmenopausal bleeding for two months (figure 1).

AFB: acid-fast bacilli

### Clinical Findings

On admission physical examination, vital signs were within normal parameters. The initial gynecological physical examination was nonspecific and showed no relevant alterations. The rest of the physical examination showed no pathological findings.

### Diagnostic evaluation

Laboratory studies evidenced moderate anemia. Transvaginal ultrasound revealed asymmetric endometrial thickening suggestive of hyperplasia. Subsequently, a nuclear magnetic resonance imaging identified multiple polypoid lesions in the endometrium-myometrial interface, affecting less than 50% of the myometrial thickness and extending towards the endocervix, findings interpreted as suggestive of endometrial cancer.

**Figure 2:**
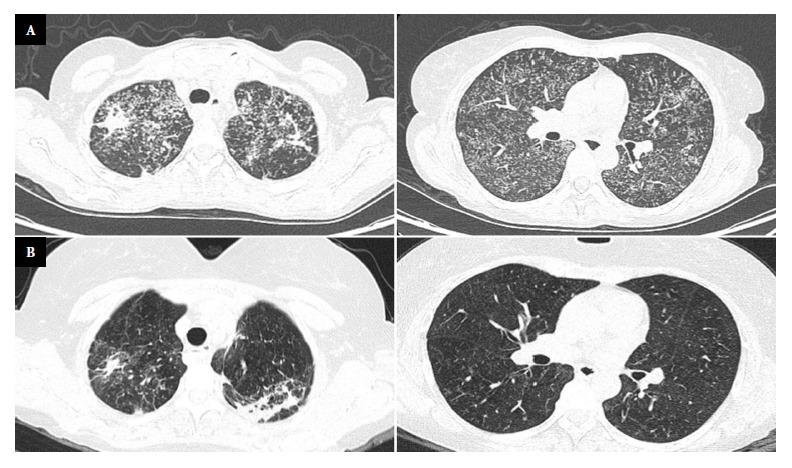
Computed tomography before and after initiation of treatment.

Given the high suspicion of gynecological neoplasia, a whole-body computed tomography with intravenous contrast was requested for oncological staging. This study showed irregular endometrial thickening with a neoplastic appearance and infiltration of adjacent structures, including periuterine fat, mesorectal fat, posterior bladder wall, and right adnexa, as well as mesenteric congestion and omental thickening suggestive of peritoneal carcinomatosis. At the pulmonary level, a bilateral centrilobular diffuse micronodular pattern and subpleural nodules in the upper lobes were observed, along with mediastinal adenopathies, the largest in a precarinal location with a maximum diameter of 9 mm, interpreted as pulmonary metastases (figure 2A).

(A) Axial images of upper sections obtained at the time of diagnosis. An irregular cavitary focus suggestive of active pulmonary tuberculosis is observed in the right upper lobe. Additionally, a diffuse micronodular pattern compatible with bronchogenic dissemination is identified in both lung parenchymas. (B) Follow-up images obtained six months after initiating therapeutic treatment. Significant resolution of the micronodular pattern and a decrease in the cavitary focus are observed. Some linear opacities and mild bronchiectasis persist, probably of a residual or cicatricial nature, without evidence of infectious activity.

Given the initial presumptive diagnosis of advanced endometrial cancer, a targeted biopsy was performed. The histopathological study revealed chronic granulomatous inflammation with caseous necrosis, presence of Langhans-type multinucleated giant cells and acid-fast bacilli (AFB) in Ziehl-Neelsen staining, thus confirming the diagnosis of multisystemic TB.

### Therapeutic intervention

The patient received standard anti-TB treatment for six months according to her body weight, consisting of two months of intensive phase with isoniazid, rifampicin, pyrazinamide, and ethambutol; followed by four months of continuation phase with isoniazid and rifampicin.

### Follow-up and results

An excellent clinical response was evidenced from the third week of treatment, with significant resolution of both respiratory and gynecological symptoms observed. Tests were performed to rule out immunocompromise such as HIV, syphilis, HTLV, hepatitis B, hepatitis C, and a rheumatological profile, all results being within normal limits. Upon completion of the therapeutic regimen, a new control endometrial biopsy was performed, which was negative for granulomas and AFB, corroborating the efficacy of the administered treatment.

### Patient's Perspective

The patient reports being very grateful to all the involved team at our hospital for the timely diagnosis of her condition, since the initial suspected diagnosis of a neoplasm had a disheartening prognosis both for her and for her family.

## DISCUSSION

FGT is established as a rare but significant clinical entity due to its potential to simulate neoplastic pathologies, especially in postmenopausal patients; however, unlike malignant processes, it constitutes a curable condition with specific treatment. The clinical presentation in our patient as suspected advanced endometrial cancer, with radiological evidence of peritoneal carcinomatosis and pulmonary metastases, emphasizes the importance of considering FGT within the differential diagnosis in cases of pelvic masses and atypical gynecological symptoms, even in the absence of obvious immunosuppression ^3^.

Extrapulmonary involvement in TB is the second most common form of presentation after lymph node involvement, with the urogenital tract being one of the most affected sites ^(^^2^. This involvement is usually the result of hematogenous dissemination from a primary pulmonary focus, although sexual transmission is also possible, which highlights the importance of a complete and multidisciplinary investigation to establish the definitive diagnosis ^10^.

**Figure 3:**
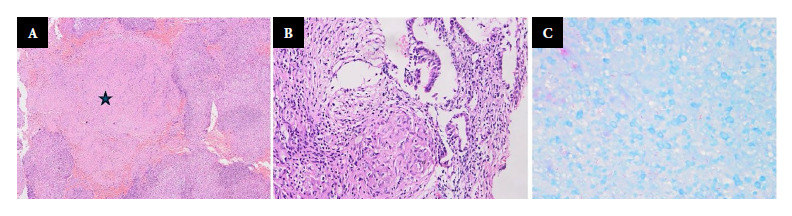
Histopathological findings in endometrial biopsy.

The histological diagnosis of the endometrial biopsy was based on the histomorphology of granulomas with caseous necrosis, presence of lymphocytic inflammatory infiltrate, epithelioid histiocytes, Langhans type multinucleated giant cells; and on histochemistry (Ziehl-Neelsen) which confirmed the presence of AFB (figure 3). However, it is important to consider that typical granulomas may be absent in some endometrial biopsies due to factors such as physiological shedding of the endometrium during menstruation ^11^. Therefore, even the isolated presence of focal lymphocytic infiltrates in endometrial biopsies should prompt diagnostic suspicion of FGT. These results underscore the importance of obtaining adequate samples and considering broader diagnostic criteria in complex clinical contexts such as the one presented.

(A) An eosinophilic central area (star), amorphous, acellular, without recognizable architecture or nuclei, is observed, which corresponds to caseous necrosis. This area is surrounded by a granulomatous inflammatory reaction. Magnification 20X. (B) Benign-looking superficial endometrial tissue is observed, adjacent to confluent epithelioid granulomas. Magnification 40X. (C) Ziehl Neelsen staining positive for acid-fast bacilli (AFB).

Standard specific treatment for six months with evident clinical response from the first weeks and subsequent negativization of the control endometrial biopsy, confirms the efficacy of internationally recommended regimens for extrapulmonary TB ^2^. The clinical, microbiological, and tomographic recovery (figure 2B) observed in the patient highlights the need for early diagnosis and timely treatment to avoid major complications, such as permanent infertility or irreversible structural damage, although in a postmenopausal patient, the focus is more on complete remission of the disease and prevention of sequelae ^(^^3^^,^^10^.

### Implications for public health

FGT continues to be a significant cause of gynecological morbidity in developing countries. As illustrated by the present case, the nonspecific clinical and imaging presentation of this pathology has the particularity of simulating advanced gynecological malignancies, which frequently leads to diagnostic confusion and a significant delay in adequate management, increasing associated morbidity. In endemic areas, this delay is exacerbated because the absence of immunosuppression often erroneously decreases clinical suspicion. To avoid late diagnoses, major sequelae, and the potential submission to unnecessary oncological interventions, it is fundamental for public health to promote continuous training of medical personnel, aimed at maintaining a high level of suspicion for extrapulmonary tuberculosis. Likewise, the integration of interdisciplinary diagnostic approaches should be established as a priority measure to guarantee the obtainment of an early histological diagnosis in the face of atypical clinical presentations or suspicion of malignancy.

The following limitations must be recognized: due to being the description of a single case, the atypical clinical presentation and the observed therapeutic response cannot be generalized to the population of postmenopausal patients with suspected gynecological neoplasia. On the other hand, although the diagnosis was solidly supported by the histopathological finding of granulomas with caseous necrosis and positivity for AFB using Ziehl-Neelsen stain, the performance of mycobacterial cultures or molecular biology tests in the endometrial tissue sample is not reported. These tests constitute the current standard for typifying the species and determining the antimicrobial susceptibility profile. Finally, the documented clinical follow-up covers the period corresponding to the six months of standard treatment and the negativization of the control biopsy, which restricts the possibility of evaluating the long-term prognosis and ruling out late relapses of the disease.

In conclusion, this clinical case contributes to medical knowledge on genital TB, emphasizing that said pathology should be kept in consideration, especially in TB-endemic regions, when facing clinical presentations of neoplastic suspicion in immunocompetent patients. The integration of interdisciplinary diagnostic approaches and adequate treatment represents a key strategy to improve the prognosis of these patients. Therefore, it is fundamental for public health that medical training be promoted to strengthen the diagnostic suspicion of extrapulmonary forms of TB.
