# Supplementary material for: Disseminated endometrial tuberculosis simulating endometrial cancer in an immunocompetent patient: a case report
Source: Rev Peru Med Exp Salud Publica. 2026 Jun 24;43(2):273–7. doi: 10.17843/rpmesp.2026.432.15677 (PMC13456915; doi:10.17843/rpmesp.2026.432.15677)
Supplement: Translation

# Translation: Tuberculosis endometrial diseminada simulando cáncer de endometrio en una paciente inmunocompetente: un reporte de caso

**Authors:** Juana V. Vera-Vera, Cristhian K. Landa-Huaman, Melissa A. Romero-Aliaga, Fernando R. Garayar-Burneo

**Affiliations:** 1 Hospital Nacional Edgardo Rebagliati Martins, Lima, Perú.

**Keywords:** Tuberculosis de los Genitales Femeninos, Hemorragia Uterina, Posmenopausia, Neoplasias Endometriales, Diagnóstico Diferencial, Tuberculosis Pulmonar

## Abstract

Se presenta el caso de una mujer de 58 años, inmunocompetente y sin antecedentes relevantes, con dolor pélvico y sangrado posmenopáusico. Los estudios de imágenes revelaron hallazgos compatibles con cáncer endometrial diseminado, incluyendo lesiones pulmonares sospechosas de metástasis por lo que inicialmente se presumió un cáncer ginecológico avanzado; sin embargo, la biopsia endometrial mostró granulomas necrotizantes con bacilos ácido-alcohol resistentes, confirmando tuberculosis (TB) endometrial. Asimismo, se evidenció compromiso pulmonar por TB (baciloscopía de esputo positiva), estableciendo el diagnóstico final de TB diseminada simulando un cáncer ginecológico. La paciente recibió tratamiento anti-TB estándar por seis meses, con remisión completa de los síntomas. Una biopsia de control postratamiento resultó negativa para TB. Este caso enfatiza la importancia de considerar la TB genital en el diagnóstico diferencial de neoplasias ginecológicas, incluso en pacientes posmenopáusicas inmunocompetentes, especialmente en regiones endémicas de TB, a fin de asegurar un diagnóstico y manejo oportuno.

## INTRODUCCIÓN

La tuberculosis (TB) extrapulmonar constituye aproximadamente el 10 al 20% de los casos de TB en pacientes inmunocompetentes. Esta proporción aumenta notablemente en presencia de inmunosupresión, llegando a más del 60% en casos de coinfección por VIH. Dentro de las formas extrapulmonares, la afectación ganglionar es la más común, seguida de la TB genitourinaria ^1^^-^^3^.

La tuberculosis genital femenina (TGF), aunque poco común en países desarrollados, continúa siendo una causa importante de morbilidad ginecológica en países en desarrollo, y es un diagnóstico relevante por sus implicancias clínicas, incluyendo infertilidad secundaria, dolor pélvico crónico, trastornos menstruales y síntomas inespecíficos como sangrado posmenopáusico ^(^^4^^,^^5^.

La TGF generalmente se origina por la diseminación hematógena del *Mycobacterium tuberculosis* desde un foco primario pulmonar hacia los órganos reproductivos femeninos. Los sitios más afectados son las trompas de Falopio en casi la totalidad de los casos, del 90 al 100%, y el endometrio en un 50 a 80% de los pacientes. Es menos habitual que la infección afecte a los ovarios, el cuello uterino o la vagina ^4^^,^^6^. Aunque es más común en mujeres jóvenes en edad reproductiva, la enfermedad puede manifestarse a cualquier edad, incluso en mujeres posmenopáusicas previamente expuestas a la primoinfección tuberculosa en etapas tempranas de su vida. En pacientes mayores inmunocompetentes, la TGF habitualmente se relaciona con la reactivación endógena de una infección latente adquirida durante la juventud ^(^^7^.

La presentación clínica suele ser inespecífica e insidiosa, dificultando notablemente el diagnóstico oportuno. Por este motivo, el diagnóstico suele establecerse de manera incidental durante evaluaciones por infertilidad, o durante la investigación diagnóstica de cuadros clínicos que simulan procesos malignos ginecológicos ^4^. De hecho, una particularidad clínica relevante es la habilidad de la TGF para simular neoplasias ginecológicas, especialmente cáncer endometrial o de ovario, dada su presentación clínica y de imágenes con masas anexiales, engrosamiento endometrial, carcinomatosis peritoneal y ascitis ^4^^,^^6^^,^^8^. Esta confusión diagnóstica puede conducir a un retraso significativo en el manejo adecuado, incrementando la morbilidad asociada a esta entidad.

Otro aspecto importante que considerar es la ocurrencia de TGF en pacientes inmunocompetentes. Aunque clásicamente se asocia TB diseminada o extrapulmonar con estados de inmunodeficiencia marcados (VIH, tratamiento inmunosupresor prolongado, entre otros), aproximadamente el 10 al 20% de casos extrapulmonares ocurren en individuos sin factores evidentes de inmunosupresión ^3^^,^^9^. Por lo tanto, la ausencia de inmunosupresión no debe excluir la sospecha diagnóstica de TB genital, particularmente en zonas endémicas.

Se describe un caso clínico de TB endometrial diseminada en una paciente inmunocompetente, con diagnóstico inicial de cáncer de endometrio avanzado. Se discuten los desafíos diagnósticos y terapéuticos de esta entidad clínica poco común, resaltando la importancia del diagnóstico histológico temprano y la relevancia clínica de considerar esta enfermedad dentro del diagnóstico diferencial en pacientes con sospecha inicial de neoplasia ginecológica maligna.

## REPORTE DE CASO

### Información de la paciente

Paciente mujer de 58 años, natural y residente del distrito de Lince en Lima, con antecedentes gineco-obstétricos de dos partos a término (G2P2002), menopausia instaurada hace ocho años sin historia previa de alteraciones menstruales, y sin antecedentes patológicos de relevancia, quien acudió a consulta por presentar dolor pélvico y sangrado posmenopáusico persistente durante dos meses (figura 1).

**Figura 1:** Línea de tiempo del relato cronológico

BAAR: bacilos ácido-alcohol resistentes.

### Hallazgos clínicos

Al examen físico de ingreso, los signos vitales se encontraron dentro de parámetros normales. El examen físico ginecológico inicial fue inespecífico y no mostró alteraciones relevantes. El resto del examen físico no mostró hallazgos patológicos.

### Evaluación diagnóstica

Los estudios de laboratorio evidenciaron anemia moderada. La ecografía transvaginal reveló un engrosamiento endometrial asimétrico sugestivo de hiperplasia. Posteriormente, una resonancia magnética nuclear identificó múltiples lesiones polipoides en la interfaz endometrio-miometrial, afectando menos del 50% del espesor del miometrio y extendiéndose hacia el endocérvix, hallazgos interpretados como sugestivos de cáncer de endometrio.

Ante la elevada sospecha de neoplasia ginecológica, se solicitó una tomografía axial computarizada corporal con contraste endovenoso para estadificación oncológica. Dicho estudio mostró engrosamiento endometrial irregular con aspecto neoplásico e infiltración de estructuras adyacentes, incluyendo grasa periuterina, mesorrectal, pared posterior vesical y anexo derecho, así como congestión mesentérica y engrosamiento omental sugerente de carcinomatosis peritoneal. A nivel pulmonar, se observó un patrón micronodular difuso centrolobulillar bilateral y nódulos subpleurales en lóbulos superiores, junto con adenopatías mediastínicas, la mayor en localización precarinal con diámetro máximo de 9 mm, interpretándose como metástasis pulmonares (figura 2A).

**Figura 2:** Tomografía axial computarizada antes y después de inicio de tratamiento.

(A) Imágenes axiales de cortes superiores obtenidad al momento del diagnóstico. Se observa en el lóbulo superior derecho un foco cavitario irregular, sugestivo de tuberculosis pulmonar activa. Además, en ambos parénquimas se identifica un patrón micronodular difuso, compatible con diseminación broncógena. (B) Imágenes de seguimiento obtenidas seis meses después de iniciar tratamiento terapéutico. Se aprecia resolución significativa del patrón micronodular disminución del foco cavitario. Persisten algunas opacidades lineales y bronquiectasias leves, probablemente de carácter residual o cicatricial, sin evidencia de actividad infecciosa.

Dado el diagnóstico presuntivo inicial de cáncer de endometrio avanzado, se realizó la biopsia dirigida. El estudio anatomopatológico reveló inflamación granulomatosa crónica con necrosis caseosa, presencia de células gigantes multinucleadas tipo Langhans y bacilos ácido-alcohol resistentes (BAAR) en tinción de Ziehl-Neelsen, confirmándose así el diagnóstico de TB multisistémica.

### Intervención terapéutica

La paciente recibió tratamiento anti-TB estándar durante seis meses según su peso corporal, que consiste en dos meses de fase intensiva con isoniazida, rifampicina, pirazinamida y etambutol; seguida de cuatro meses de fase de continuación con isoniazida y rifampicina.

### Seguimiento y resultados

Se evidenció excelente respuesta clínica desde la tercera semana de tratamiento, observándose resolución significativa de los síntomas tanto respiratorios como ginecológicos. Se realizaron pruebas para descartar inmunocompromiso como VIH, sífilis, HTLV, hepatitis B, hepatitis C y perfil reumatológico, resultando todas dentro de límites normales. Al finalizar el esquema terapéutico, se efectuó una nueva biopsia endometrial de control que resultó negativa para granulomas y BAAR, corroborando la eficacia del tratamiento administrado.

### Perspectiva de la paciente

La paciente refiere estar muy agradecida con todo el equipo involucrado de nuestro hospital por el diagnóstico oportuno de su padecimiento, puesto que la primera sospecha diagnóstica de una neoplasia era de un pronóstico desalentador tanto para ella como para su familia.

## DISCUSIÓN

La TGF se configura como una entidad clínica poco frecuente pero significativa debido a su potencial de simulación de patologías neoplásicas, especialmente en pacientes posmenopáusicas; no obstante, a diferencia de los procesos malignos, constituye una condición curable con el tratamiento específico. La presentación clínica en nuestra paciente como sospecha de cáncer endometrial avanzado, con evidencia radiológica de carcinomatosis peritoneal y metástasis pulmonares, enfatiza la importancia de considerar la TGF dentro del diagnóstico diferencial en casos de masas pélvicas y síntomas ginecológicos atípicos, aún en ausencia de inmunosupresión evidente ^3^.

La afectación extrapulmonar en la TB es la segunda forma más común de presentación después del compromiso ganglionar, siendo el tracto urogenital uno de los sitios más afectados ^(^^2^. Este compromiso suele ser resultado de una diseminación hematógena desde un foco pulmonar primario, aunque también es posible la transmisión sexual, lo cual resalta la importancia de una investigación completa y multidisciplinaria para establecer el diagnóstico definitivo ^10^.

El diagnostico histológico de la biopsia endometrial se basó en la histomorfología de granulomas con necrosis caseosa, presencia de infiltrado inflamatorio linfocitario, histiocitos epitelioides, células gigantes multinucleadas tipo Langhans; y en la histoquímica (Ziehl-Neelsen) que confirmo la presencia de BAAR (figura 3). Sin embargo, es importante considerar que los granulomas típicos pueden estar ausentes en algunas biopsias endometriales debido a factores como la descamación fisiológica del endometrio durante la menstruación ^11^. Por ello, incluso la presencia aislada de infiltrados linfocitarios focales en biopsias endometriales debe motivar la sospecha diagnóstica de TGF. Estos resultados subrayan la importancia de obtener muestras adecuadas y considerar criterios diagnósticos más amplios frente a contextos clínicos complejos como el presentado.

**Figura 3:** Hallazgos histopatológicos en biopsia endometrial.

(A) Se aprecia una zona central eosinofílica (estrella), amorfa, acelular, sin arquitectura ni núcleos reconocibles, lo que corresponde a una necrosis caseosa. Dicha área se encuentra rodeada de una reacción inflamatoria de aspecto granulomatoso. Aumento 20X. (B) Se observa tejido endometrial superficial de aspecto benigno, adyacente con granulomas epitelioides confluentes. Aumento 40X. (C) Coloración Ziehl Neelsen positivo para bacilos ácido-alcohol resistentes (BAAR).

El tratamiento específico estándar por seis meses con respuesta clínica evidente desde las primeras semanas y negativización posterior de la biopsia endometrial control, confirma la eficacia de los esquemas recomendados internacionalmente para TB extrapulmonar ^2^. La recuperación clínica, microbiológica y tomográfica (figura 2B) observada en la paciente resalta la necesidad de un diagnóstico temprano y tratamiento oportuno para evitar complicaciones mayores, como infertilidad permanente o daño estructural irreversible, aunque en una paciente posmenopáusica el enfoque se dirige más a la remisión completa de la enfermedad y prevención de secuelas ^(^^3^^,^^10^.

### Implicancias para la salud pública

La TGF continúa siendo una causa importante de morbilidad ginecológica en países en vías de desarrollo. Como lo ilustra el presente caso, la presentación clínica e imagenológica inespecífica de esta patología tiene la particularidad de simular neoplasias malignas ginecológicas avanzadas, lo cual frecuentemente conduce a una confusión diagnóstica y a un retraso significativo en el manejo adecuado, incrementando la morbilidad asociada. En zonas endémicas, este retraso se ve exacerbado porque la ausencia de inmunosupresión suele disminuir erróneamente la sospecha clínica. Para evitar diagnósticos tardíos, secuelas mayores y el potencial sometimiento a intervenciones oncológicas innecesarias, es fundamental para la salud pública promover la formación continua del personal médico, orientada a mantener un alto nivel de sospecha de tuberculosis extrapulmonar. Asimismo, se debe establecer como medida prioritaria la integración de abordajes diagnósticos interdisciplinarios que garanticen la obtención de un diagnóstico histológico temprano frente a cuadros clínicos atípicos o sospecha de malignidad.

Se deben reconocer las siguientes limitaciones: por tratarse de la descripción de un caso único, la presentación clínica atípica y la respuesta terapéutica observada no pueden generalizarse a la población de pacientes posmenopáusicas con sospecha de neoplasia ginecológica. Por otro lado, aunque el diagnóstico se sustentó sólidamente en el hallazgo histopatológico de granulomas con necrosis caseosa y la positividad para BAAR mediante la tinción de Ziehl-Neelsen, no se reporta la realización de cultivos para micobacterias ni pruebas de biología molecular en la muestra de tejido endometrial. Estas pruebas constituyen el estándar actual para tipificar la especie y determinar el perfil de sensibilidad antimicrobiana. Finalmente, el seguimiento clínico documentado abarca el periodo correspondiente a los seis meses de tratamiento estándar y la negativización de la biopsia de control, lo cual restringe la posibilidad de evaluar el pronóstico a largo plazo y descartar recaídas tardías de la enfermedad.

En conclusión, este caso clínico aporta al conocimiento médico sobre TB genital, subrayando que dicha patología debe mantenerse en consideración, especialmente en regiones endémicas de TB, ante cuadros clínicos de sospecha neoplásica en pacientes inmunocompetentes. La integración de abordajes diagnósticos interdisciplinarios y tratamiento adecuado representa una estrategia clave para mejorar el pronóstico de estos pacientes. Por lo tanto, es fundamental para la salud pública que se promueva la capacitación médica para fortalecer la sospecha diagnóstica de las formas extrapulmonares de la TB.
